# Preserving of Postnatal Leptin Signaling in Obesity-Resistant Lou/C Rats following a Perinatal High-Fat Diet

**DOI:** 10.1371/journal.pone.0162517

**Published:** 2016-09-12

**Authors:** Anne-Laure Poher, Denis Arsenijevic, Mohamed Asrih, Abdul G. Dulloo, François R. Jornayvaz, Françoise Rohner-Jeanrenaud, Christelle Veyrat-Durebex

**Affiliations:** 1 Department of Cell Physiology and Metabolism, Faculty of Medicine, University of Geneva, Geneva, Switzerland; 2 Laboratory of Metabolism, Department of Medicine Specialties, Faculty of Medicine, University of Geneva, Geneva, Switzerland; 3 Department of Medicine/Physiology, University of Fribourg, Fribourg, Switzerland; 4 Service of Endocrinology, Diabetes and Metabolism, Lausanne University Hospital, Lausanne, Switzerland; University College London, UNITED KINGDOM

## Abstract

Physiological processes at adulthood, such as energy metabolism and insulin sensitivity may originate before or weeks after birth. These underlie the concept of fetal and/or neonatal programming of adult diseases, which is particularly relevant in the case of obesity and type 2 diabetes. The aim of this study was to determine the impact of a perinatal high fat diet on energy metabolism and on leptin as well as insulin sensitivity, early in life and at adulthood in two strains of rats presenting different susceptibilities to diet-induced obesity. The impact of a perinatal high fat diet on glucose tolerance and diet-induced obesity was also assessed. The development of glucose intolerance and of increased fat mass was confirmed in the obesity-prone Wistar rat, even after 28 days of age. By contrast, in obesity-resistant Lou/C rats, an improved early leptin signaling may be responsible for the lack of deleterious effect of the perinatal high fat diet on glucose tolerance and increased adiposity in response to high fat diet at adulthood. Altogether, this study shows that, even if during the perinatal period adaptation to the environment appears to be genetically determined, adaptive mechanisms to nutritional challenges occurring at adulthood can still be observed in rodents.

## Introduction

Several evidences suggest that environmental factors modulating physiological processes at adulthood, such as energy metabolism and related insulin sensitivity may originate before or weeks after birth (i.e. the perinatal period) [1]. This raised the concept of fetal/neonatal origin or programming of adult disease, particularly with regard to obesity and type 2 diabetes, together with related cardiovascular diseases [2]. Among molecular mechanisms implicated, it seems that the main one is linked to epigenetic processes, either increased or impaired in mothers by different environmental factors such as diet, and then transmitted transiently or inter-generation (see for review [3]). The pathogenesis would also be based on altered genetic adaptation to environmental changes during development, such as those occurring during modification of the mother’s diet. Indeed, many studies in rodents reported that high fat feeding by pregnant or lactating females induced glucose intolerance and development of obesity in the progeny during adult life [4, 5] (for recent review, see [6]). Maternal high fat feeding also significantly altered the hypothalamic expression of key genes encoding for proteins involved in the regulation of food intake and energy homeostasis, such as the neuropeptide Y (NPY) [7], and in many case, the proopiomelanocortin (POMC) system [8, 9]. Importantly, maternal high fat feeding also rapidly induced leptin resistance in the arcuate nucleus and increased the susceptibility to develop diet-induced obesity in response to a high fat diet during adulthood [10, 11]. In contrast, maternal undernutrition (i.e. food restriction of 50% from embryonic day E14 to postnatal day PND21) was reported to induce a brown-like phenotype of gonadal white adipocytes until 30 days of age, suggesting that it may have long-term consequences on energy metabolism in adult rodents [12].

The Lou/C rat, an inbred strain of Wistar origin [13], was described as being resistant to the development of obesity with age or in response to a high fat (HF) diet, compared to Wistar rats [14, 15]. Such a phenotype is accompanied by a lower body fat mass [16, 17], low circulating leptin levels [16–18], increased central leptin sensitivity [19] and enhanced glucose tolerance, as well as insulin sensitivity compared to the Wistar group [16]. Interestingly, recent data also underlined a brown-like phenotype of some adipocytes in the inguinal white adipose tissue depot in Lou/C rats [20].

The first aim of this study was to determine the impact of a perinatal high fat (pHF) diet on the regulation of energy metabolism in 28 day-old Wistar and Lou/C rats. The second aim was to investigate the overall effects of a pHF diet on the development of glucose intolerance and diet-induced obesity at adulthood in the two strains of animals. To address these issues, three experiments were designed to study some of the metabolic effects of: 1) pHF diet (from embryonic day E14 to postnatal day PND28) in Wistar and Lou/C rats from birth to day 28 (PND0 to PND28); 2) pHF diet in adult Wistar and Lou/C rats submitted to a standard diet; 3) pHF diet in adult Wistar and Lou/C rats challenged with 5 weeks of high fat diet at adulthood.

## Materials and Methods

### Ethics Statement

All procedures were performed in accordance with and approved by the Institutional ethical Committee of Animal Care in Geneva and Cantonal Veterinary Office (experiment ID 1034/3684/2).

### Animals

Three month-old male and female Wistar and Lou/C rats were purchased from Charles River (L’Arbresle, France) and Harlan UK Limited (Oxon, UK), respectively. They were housed under controlled temperature (22°C) and lighting (light on: 07:00am-07:00pm) with free access to water and to a standard laboratory diet, RM3 (3.64 kcal/g) (SDS, Essex, UK) (Table 1). After one week of handling, females Wistar and Lou/C they were randomly distributed in two groups, either fed with the standard diet or with a 40% high fat diet (4.31 kcal/g) (2154 KLIBA NAFAG high fat purified diet, Provimi Kliba AG, Kaiseraugst, Switzerland) for one week only (acclimation period) (Table 1). One week later, females were placed into individual cages and bred with males for a maximum of 7 days. Pregnancies were confirmed by the presence of vaginal plugs. High fat-fed females were then kept under HF diet from the last 7 days of pregnancy (corresponding to embryonic day E14) to weaning at perinatal day 21 (PND21). High fat-fed pups (males and females) were kept under high fat diet until PND28. This protocol of high fat diet presentation was chosen as the development of hypothalamic systems regulating food intake and metabolic homeostasis is taking place during this critical late gestational period (E14 to E21/22) and in postnatal [21, 22].

**Table 1:** Composition of standard RM3 diet and high fat (HF) Kliba Nafag diet.

Ingredients (% of grams)	RM3 Std diet	2154 HF diet
Crude oil	2.60	2.50
Pork fat	-	17.50
Corn starch	61.50	15.00
Sucrose	-	26.00
Sugar (N.D.)	6.50	-
Crude protein	14.70	20.00
Maltodextrin	N.A.	10.00
Cellulose	4.32	5.00
Trace element Mix	3.60	3.70

N.D.: non defined; N.A.: non available.

After delivery, litter size was minimally adjusted to 6/8 pups/litter for Lou/C and to 8 pups/litter for Wistar. However, only male pups were considered for data analyses. One pup per litter was used.

**Fig 1:**
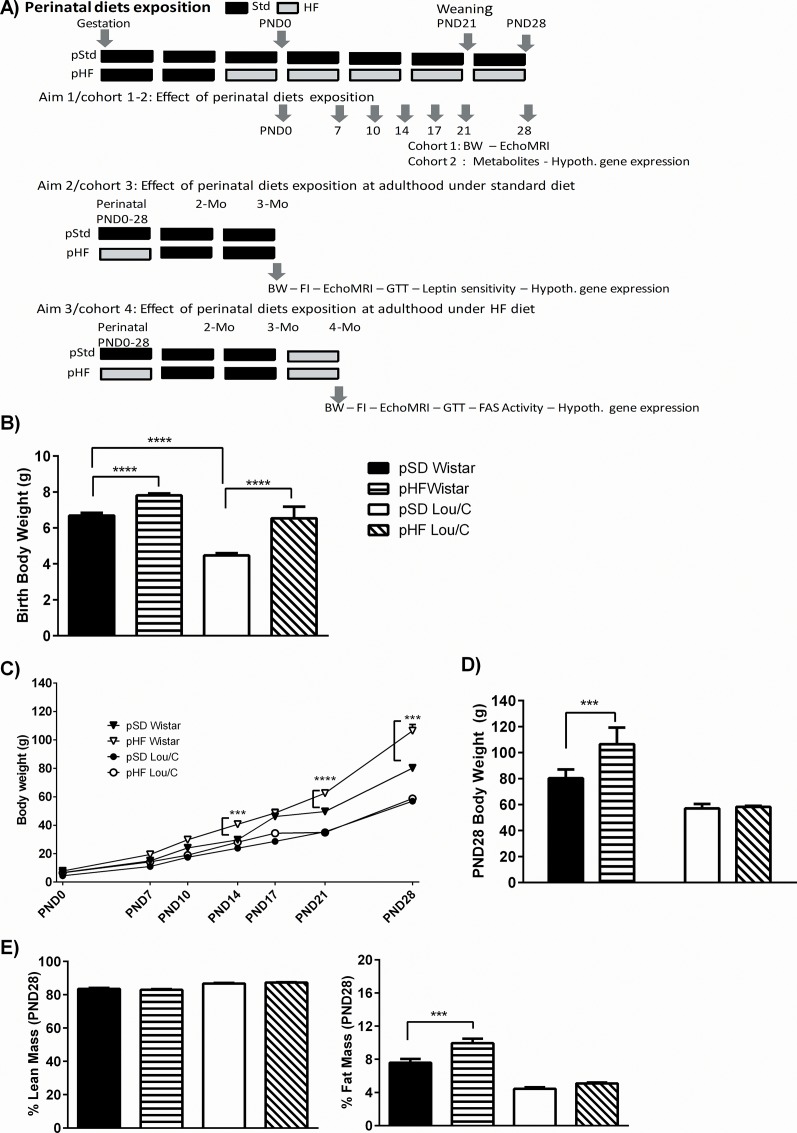
Effect of a perinatal HF diet on body weight and body composition in Wistar and Lou/C male rats. A) Diagram of perinatal diets exposition and of the 3 aims of this study. B) Body weight at birth in grams. C) Body weight gain from PND0 to PND28 in grams. D) Body weight in grams at PND28. E) Body composition expressed as percent lean and fat mass at PND28. Values are mean ± SEM (n = 8 for pSD Wistar; n = 8 for pHF Wistar; n = 8 for pSD Lou/C; n = 7 for pHF Lou/C). *p<0.05, ***p<0.001 and ****p<0.0001 *vs*. strain-related pSD rats using a two-way ANOVA.

For metabolic parameters measured in aim 1, a first cohort (cohort 1) of perinatal standard- (pSD) and high fat-fed (pHF) Wistar and Lou/C males were followed from birth to PND28. For hypothalamic gene expression and blood parameters, a second cohort (cohort 2) was euthanized at PND0, PND7, PND10, PND14, PND17, PND21, and PND28 (Fig 1A). For aims 2 and 3, sexing of male and female rats was performed at PND28. Both pSD and pHF males were fed for 2 months with the standard laboratory diet RM1 (SDS, Essex, UK). A first cohort (cohort 3) was studied at the age of 3 months under standard diet (Aim 2). A second one (cohort 4) was submitted to 5 weeks of high fat diet (HFD) at the age of 3 months, and then studied for metabolism at 4 months (Aim 3). At the end of the experiments, male rats were euthanized using isoflurane anesthesia (Halocarbon Laboratories, River Edge, NJ) and rapid decapitation. Trunk blood was sampled and tissues were rapidly removed, freeze-clamped and stored at –80°C.

### Body composition

An EchoMRI-700™ quantitative nuclear magnetic resonance analyzer (Echo Medical Systems, Houston, TX) was used to measure body composition (total fat and lean body mass).

### Peripheral (i.p.) leptin injection

Intraperitoneal (i.p.) injections of human leptin (2 mg/kg; Bachem, Bubendorf, Switzerland) or vehicle (NaCl 0.9%) were administered 1h before the start of the dark cycle (06:00pm) [19]. The anorexigenic effect of leptin was thus determined by removing the food from the cages just before leptin injection (06:00pm), and re-introducing pre-weighed amounts of food at 7:00pm. Food intake was thereafter measured at 0.5, 1, 2, 4 and 12 hrs after lights off.

### Glucose (GTT) tolerance test

Male rats were fasted for 4hrs (09:00am to 01:00pm). A glucose load of 1.5 g/kg was administered i.p. Blood samples were collected by tail nicking for further analyses of plasma glucose and insulin concentrations.

### Plasma measurements

Plasma glucose was measured by the glucose oxidase method (Glu, Roche Diagnostics GmbH, Rotkreuz, Switzerland). Plasma nonesterified fatty acids (NEFA) and triglycerides (TG) were determined using NEFA C (Wako Chemicals GmbH, Neuss, Germany) and BioMerieux (Marcy l’Etoile, France) commercial kits, respectively. Commercial EIAs were used to measure plasma leptin (Bertin Pharma, Montigny-le-Bretonneux, France), insulin (Mercodia, Uppsala, Sweden), fibroblast growth factor 1 (FGF21) (R&D systems Europe Ltd, Oxon, UK), and adiponectin (AdipoGen, San Diego, CA) levels.

### FAS activity

Measurements of fatty acid synthase (FAS) activity were determined from frozen tissues by spectrophotometry, as previously described [23].

### Tissue processing and analyses of gene expression

Total RNAs from hypothalami were extracted using a single-step extraction with Trizol reagent (Sigma-Aldrich, Buchs, Switzerland). RNA integrity was assessed by electrophoresis on a 1% agarose gel, and concentration was determined by spectrophotometry. A quantity of 2.5 μg of total RNA was used for reverse transcription, using random primers (Promega Corporation, Madisson, WI), dNTPs (Promega), Rnasin as RNase inhibitor (Promega), and the M-MLV-RT enzyme kit (Invitrogen, Basel, Switzerland). For quantitative PCR (qPCR), amplification of genes was performed from 12.5 ng cDNA using the SYBR® green PCR Master Mix (Applied Biosystems, Foster City, CA) and an ABI7500 machine. Primers were used at a concentration of 200 to 300 nM and results were normalized to the expression levels of the 36b4 housekeeping gene [24].

### Data analyses

Results are expressed as mean ± SEM. Gene expression was analyzed using the 2-ddCt method. Comparisons between Lou/C and Wistar rats for feeding patterns, metabolic parameters, plasma hormone levels and hypothalamic mRNA expression of neuropeptides or receptors implicated in the regulation of food intake were performed using two-way ANOVAs (effect of strain and diet) with Bonferroni as *post-hoc* test (GraphPad Prism, La Jolla, CA). Statistical significance was established at p<0.05. Number of animals used for all measurements and the number of litters represented are provided in tables and figure legends.

## Results

### Increased postnatal leptin signaling may prevent the occurrence of increased adiposity due to perinatal high fat feeding in male Lou/C rats

At birth, body weight (BW) was higher in pSD male Wistar than in pSD Lou/C rats (Fig 1B). The presence of high fat (HF) diet during the last week of gestation induced a significant increase in the birth weight of the same amplitude in both strains (F_(1, 27)_ = 22.39, p<0.0001). At weaning (PND21), the effect of the perinatal HF diet was still present on BW (F_(1, 27)_ = 245.3, p<0.0001), being more marked in Wistar (p<0.0001) than in Lou/C rats (Fig 1C). At PND28, the difference in BW was only present in Wistar rats (p<0.001) (Fig 1D). Interestingly, it was linked to a higher percent fat mass (F_(1, 27)_ = 16.63, p<0.001), without any change in lean mass, as determined using EchoMRI (Fig 1E).

**Fig 2:**
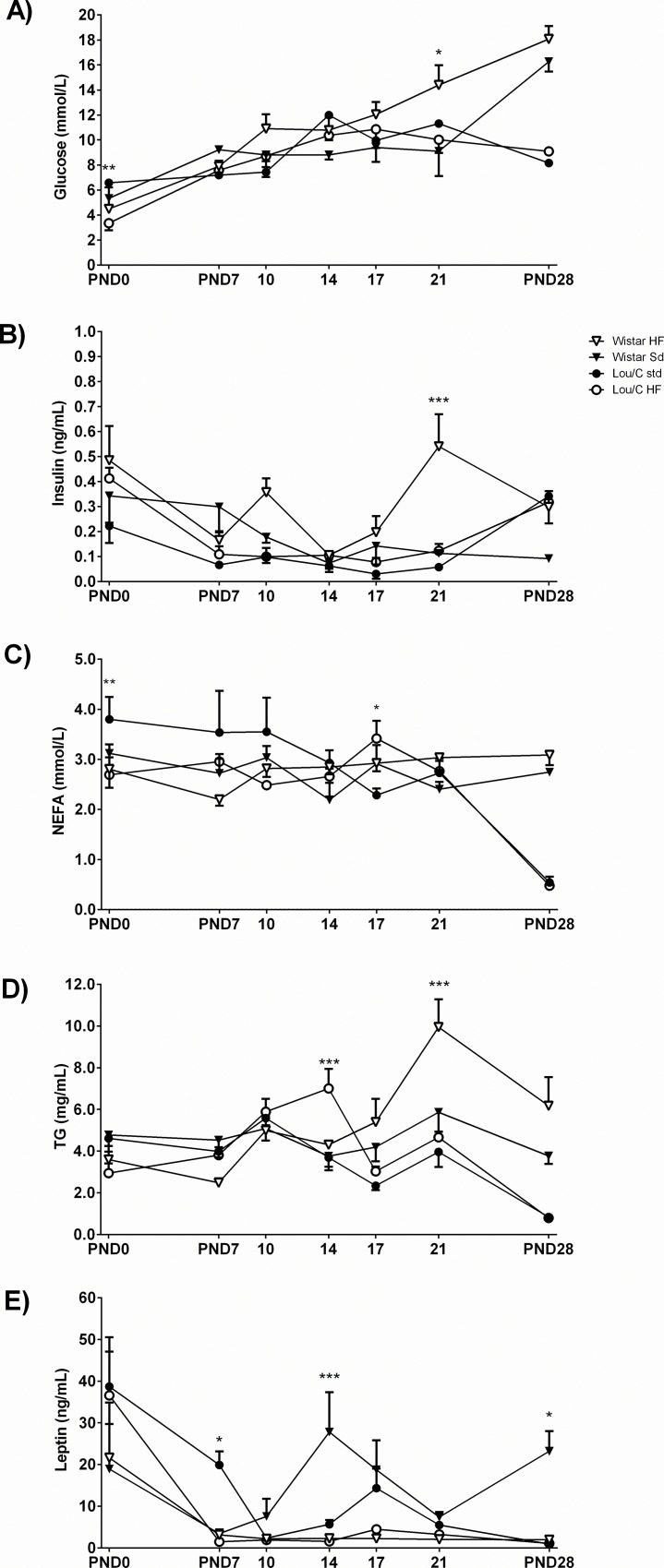
Effect of a perinatal HF diet on metabolic parameters in Wistar and Lou/C male rats from PND0 to PND28. A) Glycemia in mmol/L. B) insulinemia in ng/mL. C) Non-esterified amino acids (NEFA) in mmol/L. D) Triglycerides (TG) in mg/mL). E) Leptinemia in ng/mL. Values are mean ± SEM of 3–6 animals per group. *p<0.05 and ***p<0.001 *vs*. strain-related pSD rats using one-way ANOVA.

The profile of different metabolites and hormones was then analyzed from PND0 to PND28 in Wistar and Lou/C male rats (Fig 2). At birth, no significant difference appeared between pSD and pHF Wistar rats. In pHF Lou/C newborns, glycemia (Fig 2A) was lower and insulinemia (Fig 2B) was higher than in pSD pups. Non-esterified fatty acids (NEFA) (p<0.05) (Fig 2C) and triglycerides (TG) (p = 0.0624) (Fig 2D) were lower in pHF than in pSD Lou/C pups, suggesting a healthy metabolism, despite the presence of the perinatal HF diet. Of note, plasma leptin levels at birth were twice higher in Lou/C than in Wistar rats (Fig 2E). To our knowledge, this is the only time point at which such a hyperleptinemia is observed in this strain.

Over the 4 weeks, the HF diet significantly modified the profile of plasma TG (p<0.05), insulin (p<0.01) and leptin (p<0.05) levels in Wistar rats. A peak of TG and insulin was seen in pHF Wistar at weaning (PND21), whereas the peak of leptin observed at PND14 in pSD rats was totally blunted in pHF Wistar rats. In Lou/C animals, only plasma TG (p<0.001) and NEFA (p<0.001) profiles were slightly modified by the perinatal HF diet. In this group, the high leptin levels observed at birth were completely suppressed at PND7 in the pHF pups, while they were maintained until PND10 in pSD animals. Such leptinemia profiles in Lou/C rats may reveal the occurrence of an early postnatal increase in leptin signaling, which was only partly blunted by the pHF diet.

**Fig 3:**
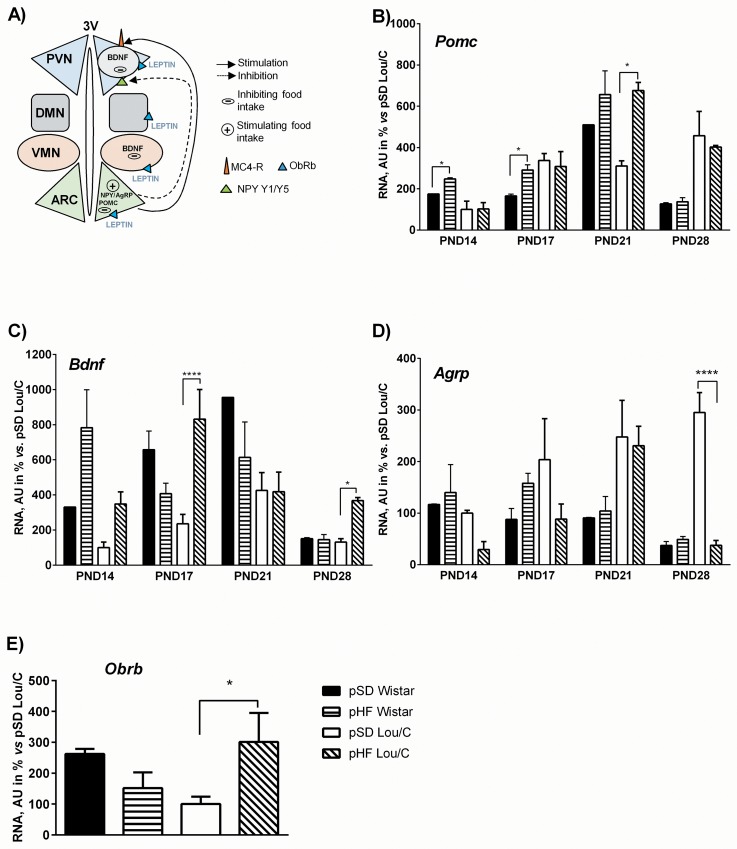
Effect of a perinatal HF diet on hypothalamic neuropeptide mRNA expression in Wistar and Lou/C male rats. A) Schematic representation of the main hypothalamic nuclei implicated in the control of food intake and metabolism. ARC: arcuate nucleus; VMN: ventromedial nucleus; DMN: dorsomedial nucleus; PVN: paraventricular nucleus. Hypothalamic mRNA expression of *Pomc* (B), *Bdnf* (C) and *Agrp* (D) in pSD (n = 3) and pHF Wistar (n = 3–4) and pSD (n = 3) and pHF Lou/C rats (n = 3–6). Values are expressed as percent of pSD Lou/C rats at PND14 (100%) and were normalized with the *36b4* mRNA expression. E) Hypothalamic mRNA expression of *Obrb*. Values are expressed as percent of pSD Lou/C rats at PND17 (100%) and were normalized with the *36b4* mRNA expression. Values are mean ± SEM (n = 3–6). *p<0.05 and ****p<0.0001 using two-way ANOVA.

Gene expression of the main hypothalamic neuropeptides, such as neuropeptide Y (NPY), proopiomelanocortin (POMC), Agouti-related peptide (AgRP) or brain-derived neurotrophic factor (BDNF), and one of the main melanocortin receptor the MC4R, all implicated in the regulation of food intake (Fig 3A), was analyzed in pSD and pHF Wistar and Lou/C male rats from PND14 to PND28. NPY is an orexigenic peptide [25], like AgRP, a specific antagonist of the most relevant melanocortin receptor, the MC4R [25]. BDNF is on the contrary an anorexigenic peptide [26, 27], like POMC [25], the precursor of αMSH. By binding to the MC4R in the paraventricular nucleus, αMSH inhibits food intake and increases energy expenditure [28]. The expression of the leptin receptor (*Obrb*) was also examined.

The perinatal HF diet did not significantly influence mRNA expressions in Wistar animals, except for *Pomc* expression that was slightly increased at PND14 and PND17 (Fig 3B). In contrast in Lou/C rats, mRNA expression of *Pomc* (p<0.05) and *Bdnf* (p<0.01) were significantly increased (Fig 3C). In pHF Lou/C rats, *Bdnf* mRNA expression was higher at PND17, whereas *Pomc* levels were higher at weaning (PND21). Finally, *Agrp* mRNA levels were drastically decreased in pHF Lou/C at PND28 (p<0.05) (Fig 3D). However, since these gene differences appeared at only one time point, it cannot be excluded that a modest shift in the timing of neurons maturation is present between the cohorts observed. Nevertheless at PND17, the *Obrb* gene expression was significantly increased in the pHF Lou/C group (Fig 3E), which might contribute to counteract the deleterious effect of the pHF diet in this strain.

**Fig 4:**
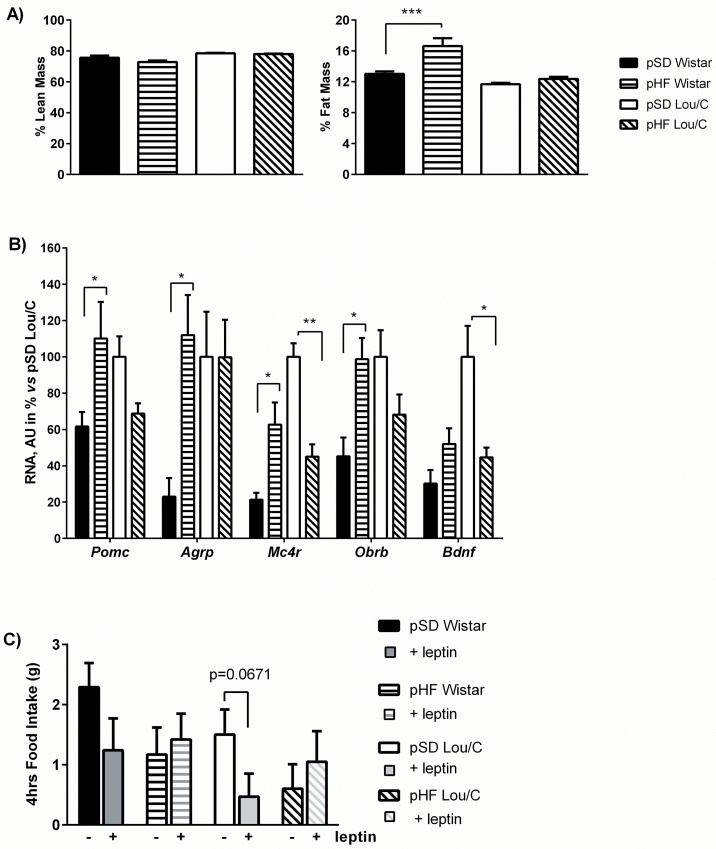
Effect of a perinatal HF diet on body composition, hypothalamic neuropeptide mRNA expression and leptin sensitivity in 3-month old Wistar and Lou/C male rats under a standard diet. A) Body composition expressed in percent lean and fat mass. Values are mean ± SEM (n = 8 for pSD Wistar; n = 8 for pHF Wistar; n = 6 for pSD Lou/C; n = 8 for pHF Lou/C). B) Hypothalamic mRNA expression of *Pomc*, *Agrp*, *Mc4r*, *Obrb* and *Bdnf* in Wistar and Lou/C rats. Values are expressed as percent of pSD Lou/C rats (100%) and were normalized with the *36b4* mRNA expression. Values are mean ± SEM (n = 4 for pSD Wistar; n = 4 for pHF Wistar; n = 4 for pSD Lou/C; n = 4 for pHF Lou/C). C) Effect of acute leptin injection (i.p., 2 mg/kg, 1h before the light off) on 4hrs-food intake. Values are mean ± SEM (n = 8 for pSD Wistar; n = 8 for pHF Wistar; n = 6 for pSD Lou/C; n = 8 for pHF Lou/C). *p<0.05, **p<0.01 and ***p<0.001 using one-way and two-way ANOVA.

At 3 months of age, no effect of the perinatal HF diet was seen on body weight of Wistar and Lou/C male rats (Table 2). The food intake was also comparable in pHF and pSD animals. However, a significant increase in the percent fat mass was observed in pHF compared to pSD Wistar rats (F_(1, 42)_ = 6.074, p<0.05), while no change was observed for the lean mass (Fig 4A). When analyzing different plasma parameters of energy metabolism, no significant difference appeared for plasma insulin and glucose levels (Table 2). Lower NEFA and leptin concentrations were detected in Lou/C *vs* Wistar rats, as already described [16]. Furthermore and as expected, the higher fat mass of pHF Wistar rats was correlated with their increased plasma leptin levels.

**Table 2:** Metabolic and hormonal parameters of adult (3 months of age) pSD and pHF Wistar and Lou/C male rats fed a standard diet at adulthood.

	pSD Wistar (n = 12)	pHF Wistar (n = 12)	pSD Lou/C (n = 8)	pHF Lou/C (n = 14)
**BW (g)**	335 ± 5	341 ± 6	256 ± 4^ab^	264 ± 5^ab^
**Food Intake (g/d)**	21.8 ± 0.3	22.1 ± 0.3	18.5 ± 0.5^ab^	18.5 ± 0.4^ab^
**HOMA Index**	20.3 ± 2.4	19.1 ± 1.6	8.3 ± 2.1^ab^	12.6 ± 2.0^ab^
**Insulin (ng/mL)**	2.3 ± 0.3	2.2 ± 0.2	2.1 ± 0.2	2.1 ± 0.4
**Leptin (ng/mL)**	3.5 ± 0.3	5.4 ± 0.3 ^a^	1.8 ± 0.2^a^	2.0 ± 0.5^ab^
**Glucose (mmol/L)**	8.0 ± 0.7	10.3 ± 1.9	6.9 ± 0.7	7.1 ± 0.6
**NEFA (mmol/L)**	1.16 ± 0.02	1.09 ± 0.03	0.62 ± 0.09^ab^	0.89 ± 0.05^ab^
**Adiponectin (μg/mL)**	33.9 ± 1.6	39.8 ± 5.3	13.1 ± 1.1^ab^	18.0 ± 1.3^ab^
**FGF21 (pg/mL)**	174 ± 29	258 ± 54	339 ± 65	282 ± 51

The HOMA index was calculated as [fasted glycemia (mmol/L) x fasted insulinemia (mUI/L)/22.5]. ^a^p<0.05 compared to pSD Wistar and ^b^p<0.05 compared to pHF Wistar, using the Student’s *t* test.

Regarding hypothalamic gene expression, significant modifications of the melanocortin system were observed in Wistar male rats (Fig 4B). Indeed, the perinatal HF diet induced a 1.8-, 4.8- and 3-fold increase in *Pomc*, *Agrp* and *Mc4r* mRNA expression, respectively. A 2.2 fold increase in *Obrb* was also observed. However, since no difference on food intake was noticed, these modifications are likely to compensate each another. In pHF Lou/C rats, only the mRNA expression of *Mc4r* and *Bdnf* was significantly decreased (Fig 4B). Once again, it seems to have no impact on the feeding pattern. Leptin sensitivity was thereafter tested by injecting human leptin (2 mg/kg), 1h before the light off. Food intake was analyzed 30 minutes, 1, 2 and 4 hrs later. As previously observed [19], no anorexigenic effect of leptin was observed 30 minutes after the lights off, being only significant after 2 and 4 hrs. In this study, a decrease of food intake was observed in pSD Lou/C rats only (Fig 4C). In other groups, no effect was noticed, suggesting the presence of a central leptin resistance. Since *Obrb* mRNA expression was increased in the pHF Wistar group, such central leptin resistance could be related to decreased intracellular leptin signaling, whereas a decreased activity of the BDNF system could mediate this process in Lou/C rats [19].

### Perinatal high fat diet induces glucose intolerance in adult Wistar, but not in Lou/C male rats

**Fig 5:**
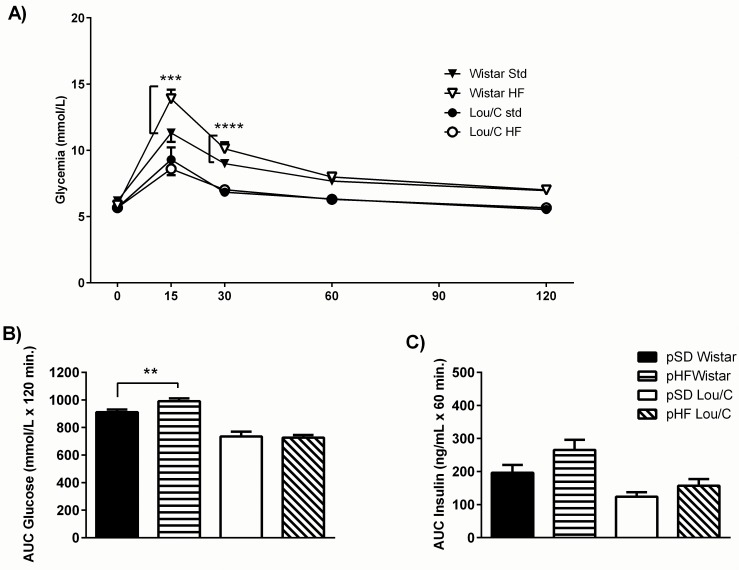
Effect of a perinatal HF diet on glucose tolerance in 3-month old Wistar and Lou/C male rats under a standard diet. A) Evolution of delta glycemia (mM) at 0, 15, 30, 60 and 120 min after acute glucose injection (1.5 g/kg, i.p.). B) Areas under the curves (AUCs) of glycemia (mmol/L x min) over 120 minutes following the glucose load. C) Areas under the curves (AUCs) of insulin (ng/mL x min) over the 60 minutes following the glucose load. Values are mean ± SEM (n = 6 for pSD Wistar; n = 6 for pHF Wistar; n = 6 for pSD Lou/C; n = 6 for pHF Lou/C). **p<0.01, ***p<0.001 and ****p<0.0001 using the two-way ANOVA.

To further characterize the metabolic phenotype of adult rats, a GTT was performed in 4hr-fasted rats. As can be seen on Fig 5A, HF during the perinatal period significantly altered glucose tolerance in Wistar rats at 15 and 30 min. after the glucose load (F_(5, 132)_ = 29.30, p<0.0001). Moreover, the areas under the curves (AUCs) of the glycemia, 120 minutes after the glucose load was significantly higher in pHF Wistar compared to pSD rats (p<0.01) (Fig 5B). This glucose intolerance was present despite a tendency toward increased insulin release, as calculated using AUCs over 60 minutes (Fig 5C), suggesting the presence of insulin resistance in peripheral tissues. No alteration of glucose tolerance or of insulin secretion was observed in Lou/C rats.

In order to explain the occurrence of glucose intolerance in pHF Wistar rats, plasma levels of adiponectin and FGF21, two modulators of insulin sensitivity [29–32], were determined. However, none of these hormones was significantly modified in pHF compared to pSD rats (Table 2).

### Permissive effect of a perinatal high fat diet on the development of diet-induced obesity in adult Wistar, but not in Lou/C male rats

**Fig 6:**
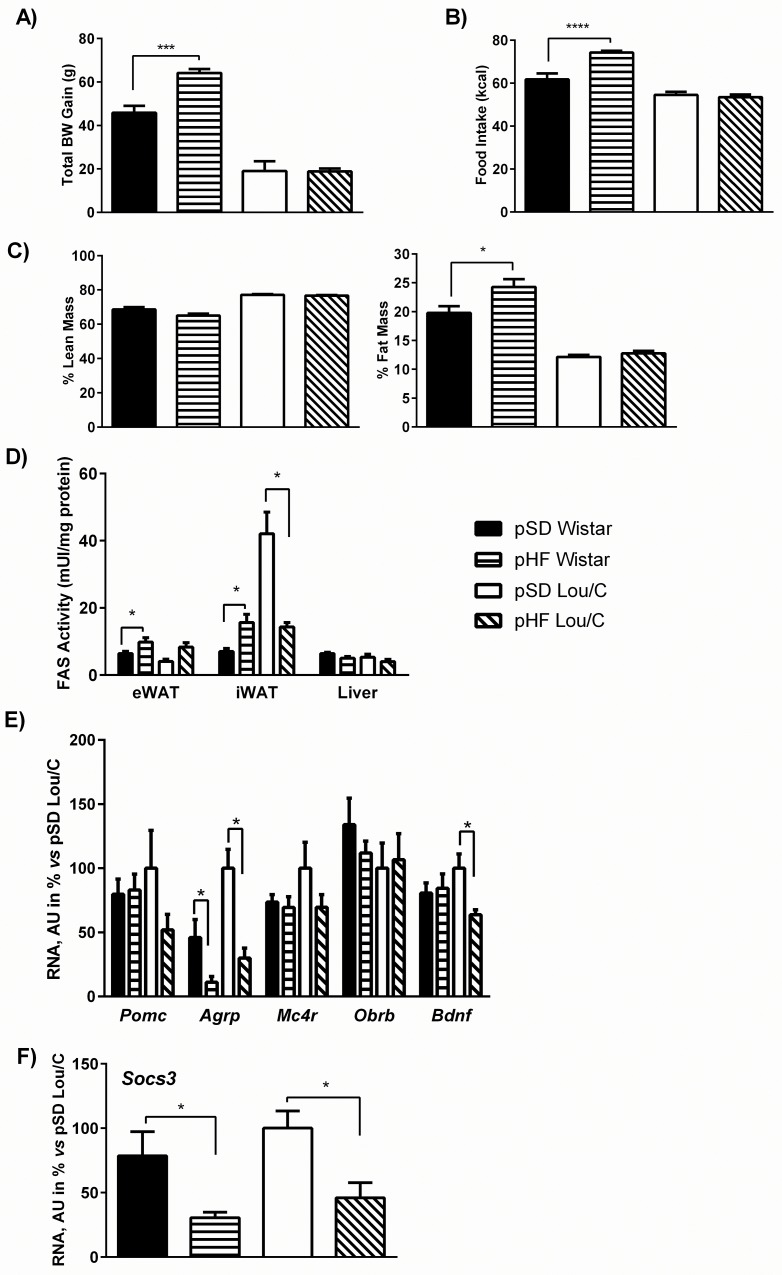
Effect of a perinatal HF diet on body weight gain, daily food intake, body composition, FAS activity and hypothalamic neuropeptide mRNA expression in 4-month old Wistar and Lou/C male rats under a high fat diet. A) Total body weight gain over the 5 weeks of high fat diet. B) Mean daily (24hrs) food intake expressed in kilocalories. C) Body composition expressed as percent lean and fat mass. D) FAS activity (in mU per mg of protein) in the liver, epididymal (eWAT) and inguinal (iWAT) white adipose tissues. Values are mean ± SEM (n = 8 for pSD Wistar; n = 8 for pHF Wistar; n = 6 for pSD Lou/C; n = 8 for pHF Lou/C). E) Hypothalamic mRNA expression of *Pomc*, *Agrp*, *Mc4r*, *Obrb* and *Bdnf*. Values are expressed as percent of pSD Lou/C rats (100%) and were normalized with the *36b4* mRNA expression. F). Hypothalamic mRNA expression of *Socs3*. Values are expressed as percent of pSD Lou/C rats (100%) and were normalized with the *36b4* mRNA expression. Values are mean ± SEM (n = 8 for pSD Wistar; n = 8 for pHF Wistar; n = 6 for pSD Lou/C; n = 7 for pHF Lou/C). *p<0.05, ***p<0.001 and ****p<0.0001 using two-way ANOVA.

In this experiment, adult Wistar and Lou/C males subjected to pSD or to pHF were submitted to a high fat diet for 5 weeks at the age of 3 months. A significantly higher BW gain over the 5 weeks of high fat diet was observed in the Wistar group (F_(1, 26)_ = 10.86, p<0.01) (Fig 6A). It was correlated with a higher caloric intake (F_(1, 26)_ = 13.89, p<0.001) (Fig 6B) and with a higher percent fat mass (F_(1, 26)_ = 28.97, p<0.0001) (Fig 6C). Accordingly, a significant stimulation of FAS activity was observed in both epididymal (eWAT) and inguinal (iWAT) white adipose tissues (Fig 6D). When analyzing plasma parameters, no significant difference appeared between Wistar and Lou/C rats (Table 3), except for plasma leptin levels, as already described [16]. Thus, leptinemia was higher in the two groups of Wistar compared to Lou/C rats (F_(1, 26)_ = 90.27, p<0.0001), with no difference between the pSD and pHF Wistar groups.

**Table 3:** Metabolic and hormonal parameters of adult (4 months of age) pSD and pHF Wistar and Lou/C male rats fed a high fat diet at adulthood.

	pSD Wistar (n = 8)	pHF Wistar (n = 8)	pSD Lou/C (n = 6)	pHF Lou/C (n = 8)
**Initial BW (g)**	392 ± 7	382 ± 11	279 ± 5^ab^	287 ± 8^ab^
**HOMA Index**	28.5 ± 2.9	36.3 ± 3.5	17.6 ± 1.9 ^ab^	19.4 ± 2.9 ^ab^
**Insulin (ng/mL)**	2.3 ± 0.2	2.9 ± 0.4	1.9 ± 0.5	2.1 ± 0.3
**Leptin (ng/mL)**	4.2 ± 0.1	4.3 ± 0.1	2.7 ± 0.3^ab^	3.2 ± 0.2^ab^
**Glucose (mmol/L)**	13.6 ± 1.5	12.0 ± 1.3	9.7 ± 0.7	11.2 ± 1.1
**NEFA (mmol/L)**	0.77 ± 0.04	0.65 ± 0.05	0.83 ± 0.08	0.98 ± 0.11^b^

The HOMA index was calculated as [fasted glycemia (mmol/L) x fasted insulinemia (mUI/L)/22.5]. ^a^p<0.05 compared to pSD Wistar and ^b^p<0.05 compared to pHF Wistar, using the Student’s *t* test.

Hypothalamic mRNA expression profiles showed that, on the contrary to what was observed under the standard diet, levels of *Pomc* and *Mc4r* were no more modified in pHF Wistar rats compared to the pSD groups. However, *Agrp* expression was significantly decreased (Fig 6E). In pHF Lou/C rats, both *Agrp* and *Bdnf* gene expression levels were decreased. Moreover, mRNA expression of *Socs3*, the transcription factor suppressor of cytokine signaling 3 [33], involved in the inhibition of leptin signaling, was decreased in both the pHF Wistar and Lou/C group, compared to their pSD counterparts (Fig 6F).

**Fig 7:**
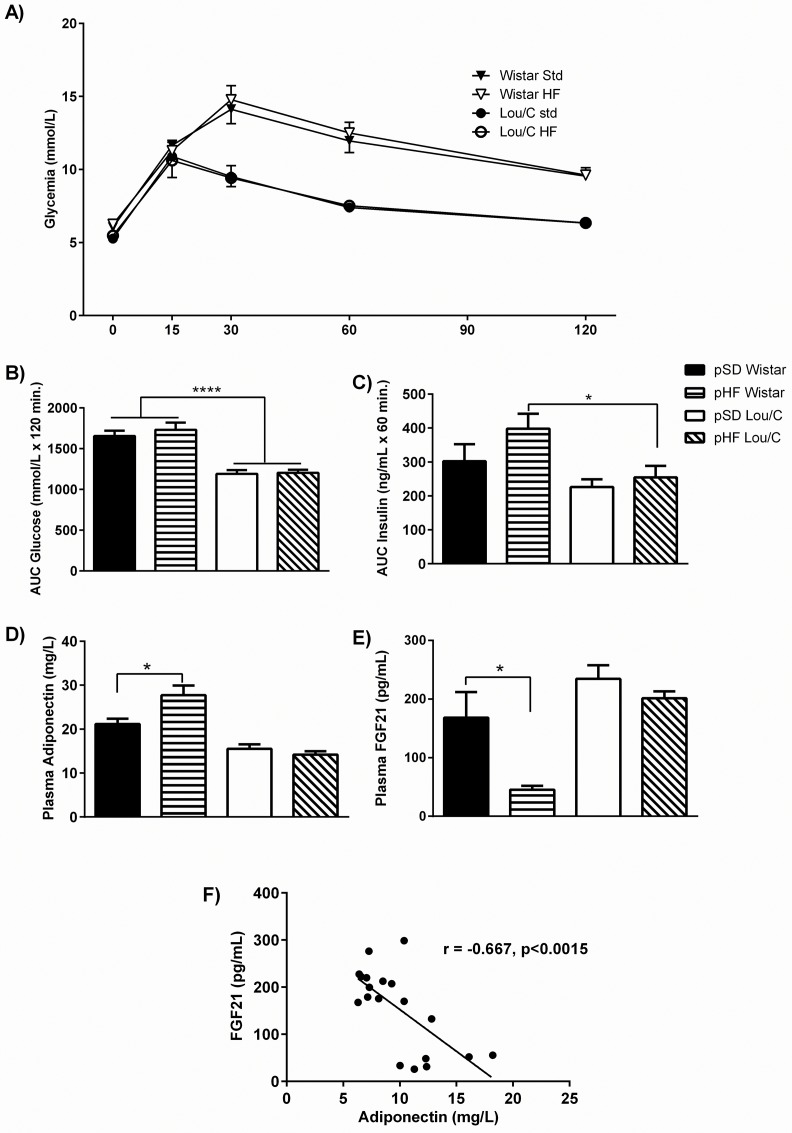
Effect of a perinatal HF diet on glucose tolerance in 4-month old Wistar and Lou/C male rats under a high fat diet. A) Evolution of delta glycemia (mM) at 0, 15, 30, 60 and 120 min after acute glucose injection (1.5 g/kg, i.p.). B) Areas under the curves (AUCs) of glycemia (mmol/L x min) over 120 minutes following the glucose load. C) Areas under the curves (AUCs) of insulin (ng/mL x min) over 60 minutes following the injection. Plasma adiponectin (D) and FGF21 (E) levels. F) Correlation between plasma adiponectin and FGF21 levels in both Wistar and Lou/C rats using Pearson r test. Values are mean ± SEM (n = 8 for pSD Wistar; n = 8 for pHF Wistar; n = 6 for pSD Lou/C; n = 8 for pHF Lou/C). *p<0.05 and ****p<0.0001 using the two-way ANOVA.

Despite the difference in adiposity, the glucose tolerance (Fig 7A and 7B), as well as the HOMA index (Table 3), were similarly altered by the 5 weeks of HFD in pSD and pHF Wistar male rats. A tendency toward an increased insulin release during the GTT was observed in the pHF Wistar group (Fig 7C). Other mechanisms involved in insulin sensitization were then investigated in Wistar rats. Interestingly, plasma levels of adiponectin were increased (F_(1, 15)_ = 5.74, p = 0.0301) (Fig 7D), while plasma FGF21 concentrations were decreased (F_(1, 15)_ = 9.72, p = 0.0066) in pHF Wistar rats (Fig 7E). An inverse correlation between these two hormonal parameters was even observed (r = -0.677, p = 0.0015) (Fig 7F). No change in any of these parameters was observed in Lou/C rats.

## Discussion

Contrary to what was previously described in male Sprague Dawley rats [34], consumption of a high fat (HF) diet during the perinatal period (including one week of HF diet before birth) induced a significant increase in birth body weight (BW) in both Wistar and Lou/C male rats. However, this difference disappeared with age in Lou/C rats, whereas it was maintained until PND28 in the Wistar group, as previously observed in this strain using a similar protocol [35]. The increased body weight of pHD Wistar rats at 28 days was exclusively due to a higher fat mass. Higher glycemia, despite hyperinsulinemia at postnatal day PND21 in pHF Wistar pups, also argues in favor of a decreased insulin sensitivity in this group. On the contrary, lower glycemia and NEFA levels and higher plasma insulin levels in pHF than pSD Lou/C rats suggested that insulin sensitivity was not altered and that Lou/C pups developed an adaptive response to the perinatal HF diet.

Interestingly, the leptin profile was notably modified in pHF Wistar rats compared to pSD pups during the postnatal period. Thus, the leptin surge described at PND10 in C57BL/6J mice [36] or in Wistar rats [37] was observed at PND14-d17 in pSD rats, but was totally blunted in pHF pups. Several studies have reported that the neonatal leptin surge plays an important role in the development of the hypothalamic circuitry involved in the regulation of food intake and adiposity [21, 36, 38, 39]. Notably, blunted leptin surge by maternal undernutrition was shown to affect arcuate *Pomc* expression and projections of POMC neurons [37]. More recently, the leptin action on the activity of NPY/AgRP/GABA neurons was also described in the periweaning period [40], comforting the idea that, probably, both POMC and NPY/AgRP neurons activities and/or densities could program later sensitivity to high caloric diet. In our study, no significant hypothalamic modifications were observed in pHF Wistar pups. However, these results should be taken with caution since the analyses were done on all the hypothalamus (not arcuate nucleus only) and no exploration of neurons projections were realized. In pSD Lou/C rats, a normal leptin surge was observed, although it occurred earlier than in pSD Wistar animals. Interestingly in the Lou/C group, the deleterious effect of the perinatal HF diet appeared to be counteracted by the presence of increased *Obrb* expression and therefore improved leptin signaling. This is the likely explanation for the observations of decreased expression of orexigenic factors and increased expression of anorexigenic ones.

Regarding the metabolic consequences of the leptin surge at adulthood, it has been shown that its blockade by administration of a leptin antagonist predisposes adult rats to increased body weight gain, higher susceptibility to the development of obesity and leptin resistance [41, 42]. In the present study, these parameters were investigated in the four groups of rats, first under a standard diet (STD), and secondly after a 5-week exposure to a high fat diet (HFD). Under STD (3 months of age), neither pHF Wistar nor pHF Lou/C rats showed modification of BW or food intake. Only the percent fat mass was significantly increased in pHF Wistar (16.6 ± 1.0 *vs*. 12.4 ± 0.3% for pSD rats, p = 0.0025). However, both pHF Wistar and Lou/C rats were resistant to the acute effect of leptin on food intake. Regarding body weight homeostasis, some discrepancies between the two strains appeared under HFD at adulthood. Indeed, increased food intake, BW and adiposity were exacerbated in pHF Wistar compared to pSD rats, confirming the higher susceptibility to the development of diet-induced obesity in this strain compared to Lou/C rats [16, 17]. Interestingly, this did not occur in Lou/C rats.

Investigating insulin sensitivity, it was shown that chronic consumption of a HF diet by pregnant and lactating female rats induced hyperinsulinemia in the litter after weaning [43], causing glucose intolerance at adulthood [4]. Indeed, at 3 months of age, under a standard diet, a significant glucose intolerance, probably linked to insulin resistance, was observed in pHF Wistar rats. Interestingly, this glucose intolerance was not exacerbated after HFD, since similar glycemia profiles were observed in pSD and pHF Wistar rats, suggesting that the deleterious effect of the pHF was obscured by the consumption of a HF diet at adulthood. Alternatively, it may be that compensatory mechanisms occurred in pHF Wistar rats to prevent a further worsening of glucose tolerance. In keeping with this hypothesis, plasma adiponectin levels were significantly increased in pHF compared to pSD Wistar rats. Another hypothesis that could also be proposed is based on the observation of increased enzymatic activity of fatty acid synthase in adipose tissue depots of Wistar rats. Indeed, it has recently been shown that channeling of glucose utilization toward *de novo* lipogenesis (DNL) can be stimulated in white adipose tissue in a rat model of semi-starvation/refeeding that exhibits catch-up fat, thus maintaining glucose homeostasis [44]. A similar process might underlie the lack of worsening of glucose tolerance in pHF *vs*. pSD Wistar animals. Of note, these putative adaptations are observed after a short exposure to HFD (5 weeks). It would be interesting to know what would happen after a longer period. Moreover, hyperinsulinemia during the perinatal period was also shown to be correlated with higher galaninergic neurons development in the ARC of neonatal overfed rats by reducing litter size, thus promoting overweight and metabolic syndrome during life [45]. It can be hypothesized that this mechanism could be involve, considering that galanin highly predispose to high-fat diet consumption [46].

In Lou/C rats, no deleterious impact of the pHF diet on glucose tolerance or diet-induced obesity was observed at adulthood. This is likely mediated by the increased postnatal leptin sensitivity observed in this strain, as mentioned above. Of note at that point is the fact that leptin’s effects during the postnatal two week period appears to be independent from its anorexigenic effect and to be mediated by increased thermogenesis linked to uncoupling protein 1 (*Ucp1*) expression [47]. Accordingly, animal models of leptin deficiency have decreased brown adipose tissue (BAT) activity [48] and leptin treatment was reported to induce thermogenesis in BAT *via* activation of the beta-adrenergic system [49, 50]. Furthermore, activation of the sympathetic nervous system was also known to promote the “browning” of subcutaneous white adipose tissue [51], which is actually one of the main characteristics of Lou/C rats [17, 52].

To sum up, this study underscores the adaptive capacity of Lou/C rats to different diet challenges, in order to maintain caloric intake, adiposity and insulin sensitivity over time probably through leptin signaling. Moreover, as previously described [1, 4–6, 34, 41, 42], perinatal HF diet alters glucose tolerance in adult Wistar rats under a standard diet, and increases the susceptibility to develop diet-induced obesity. When challenged by a HFD at adulthood, Wistar rats seem to develop temporary compensatory mechanisms, in order to avoid any exacerbation of insulin resistance. Altogether, the present data show that, although the adaptation to environmental changes during the perinatal period appears to be genetically determined in rodents, additional adaptation mechanisms to nutritional changes occurring at adulthood can still be observed.

## References

[1] Beall MH, El Haddad M, Gayle D, Desai M, Ross MG. Adult obesity as a consequence of in utero programming. Clinical obstetrics and gynecology. 2004;47(4):957–66; discussion 80–1. . 15596948 10.1097/01.grf.0000135668.61661.9c

[2] Hales CN, Barker DJ. The thrifty phenotype hypothesis. British medical bulletin. 2001;60:5–20. . 11809615 10.1093/bmb/60.1.5

[3] Martinez JA, Cordero P, Campion J, Milagro FI. Interplay of early-life nutritional programming on obesity, inflammation and epigenetic outcomes. Proc Nutr Soc. 2012;71(2):276–83. 10.1017/S0029665112000055 . 22390978

[4] Srinivasan M, Katewa SD, Palaniyappan A, Pandya JD, Patel MS. Maternal high-fat diet consumption results in fetal malprogramming predisposing to the onset of metabolic syndrome-like phenotype in adulthood. American journal of physiology Endocrinology and metabolism. 2006;291(4):E792–9. 10.1152/ajpendo.00078.2006 . 16720630

[5] Armitage JA, Taylor PD, Poston L. Experimental models of developmental programming: consequences of exposure to an energy rich diet during development. The Journal of physiology. 2005;565(Pt 1):3–8. 10.1113/jphysiol.2004.079756 15695245 PMC1464498

[6] Dyer JS, Rosenfeld CR. Metabolic imprinting by prenatal, perinatal, and postnatal overnutrition: a review. Seminars in reproductive medicine. 2011;29(3):266–76. 10.1055/s-0031-1275521 . 21769766

[7] Page KC, Malik RE, Ripple JA, Anday EK. Maternal and postweaning diet interaction alters hypothalamic gene expression and modulates response to a high-fat diet in male offspring. American journal of physiology Regulatory, integrative and comparative physiology. 2009;297(4):R1049–57. 10.1152/ajpregu.90585.2008 . 19657097

[8] Kirk SL, Samuelsson AM, Argenton M, Dhonye H, Kalamatianos T, Poston L, et al Maternal obesity induced by diet in rats permanently influences central processes regulating food intake in offspring. PloS one. 2009;4(6):e5870 10.1371/journal.pone.0005870 19516909 PMC2690656

[9] Bouret SG, Simerly RB. Development of leptin-sensitive circuits. J Neuroendocrinol. 2007;19(8):575–82. 10.1111/j.1365-2826.2007.01563.x . 17620099

[10] Glavas MM, Kirigiti MA, Xiao XQ, Enriori PJ, Fisher SK, Evans AE, et al Early overnutrition results in early-onset arcuate leptin resistance and increased sensitivity to high-fat diet. Endocrinology. 2010;151(4):1598–610. 10.1210/en.2009-1295 20194730 PMC2850236

[11] Gorski JN, Dunn-Meynell AA, Hartman TG, Levin BE. Postnatal environment overrides genetic and prenatal factors influencing offspring obesity and insulin resistance. American journal of physiology Regulatory, integrative and comparative physiology. 2006;291(3):R768–78. 10.1152/ajpregu.00138.2006 . 16614055

[12] Delahaye F, Lukaszewski MA, Wattez JS, Cisse O, Dutriez-Casteloot I, Fajardy I, et al Maternal perinatal undernutrition programs a "brown-like" phenotype of gonadal white fat in male rat at weaning. American journal of physiology Regulatory, integrative and comparative physiology. 2010;299(1):R101–10. 10.1152/ajpregu.00604.2009 . 20463183

[13] Bazin H. The Louvain (LOU) rats. Cleveland, OH: CRC Press; 1990 43–51 p.

[14] Veyrat-Durebex C, Alliot J. Changes in pattern of macronutrient intake during aging in male and female rats. Physiol Behav. 1997;62(6):1273–8. . 9383113 10.1016/s0031-9384(97)00304-1

[15] Veyrat-Durebex C, Boghossian S, Alliot J. Age-related changes in adaptive mechanisms of macronutrient self-selection: evidence for a sexual dimorphism. Mech Ageing Dev. 1998;103(3):223–34. . 9723900 10.1016/s0047-6374(98)00013-x

[16] Veyrat-Durebex C, Montet X, Vinciguerra M, Gjinovci A, Meda P, Foti M, et al The Lou/C rat: a model of spontaneous food restriction associated with improved insulin sensitivity and decreased lipid storage in adipose tissue. American journal of physiology Endocrinology and metabolism. 2009;296(5):E1120–32. Epub 2009/02/12. 90592.2008 [pii] 10.1152/ajpendo.90592.2008 . 19208855

[17] Veyrat-Durebex C, Poher AL, Caillon A, Montet X, Rohner-Jeanrenaud F. Alterations in lipid metabolism and thermogenesis with emergence of brown adipocytes in white adipose tissue in diet-induced obesity-resistant Lou/C rats. American journal of physiology Endocrinology and metabolism. 2011;300(6):E1146–57. Epub 2011/03/17. 10.1152/ajpendo.00575.2010 . 21406614

[18] Taleux N, De Potter I, Deransart C, Lacraz G, Favier R, Leverve XM, et al Lack of starvation-induced activation of AMP-activated protein kinase in the hypothalamus of the Lou/C rats resistant to obesity. Int J Obes (Lond). 2008;32(4):639–47. Epub 2007/12/07. 10.1038/sj.ijo.0803759 . 18059408

[19] Veyrat-Durebex C, Poher AL, Caillon A, Somm E, Vallet P, Charnay Y, et al Improved leptin sensitivity as a potential candidate responsible for the spontaneous food restriction of the Lou/C rat. PloS one. 2013;8(9):e73452 10.1371/journal.pone.0073452 24039946 PMC3765307

[20] Veyrat-Durebex C, Poher AL, Caillon A, Montet X, Rohner-Jeanrenaud F. Alterations in lipid metabolism and thermogenesis with emergence of brown adipocytes in white adipose tissue in diet-induced obesity-resistant Lou/C rats. American journal of physiology Endocrinology and metabolism. 2011;300(6):E1146–57. 10.1152/ajpendo.00575.2010 . 21406614

[21] Bouret SG, Draper SJ, Simerly RB. Trophic action of leptin on hypothalamic neurons that regulate feeding. Science. 2004;304(5667):108–10. 10.1126/science.1095004 . 15064420

[22] Grove KL, Grayson BE, Glavas MM, Xiao XQ, Smith MS. Development of metabolic systems. Physiol Behav. 2005;86(5):646–60. 10.1016/j.physbeh.2005.08.063 . 16289141

[23] Linn TC. Purification and crystallization of rat liver fatty acid synthetase. Archives of biochemistry and biophysics. 1981;209(2):613–9. . 7294812 10.1016/0003-9861(81)90320-9

[24] Akamine R, Yamamoto T, Watanabe M, Yamazaki N, Kataoka M, Ishikawa M, et al Usefulness of the 5' region of the cDNA encoding acidic ribosomal phosphoprotein P0 conserved among rats, mice, and humans as a standard probe for gene expression analysis in different tissues and animal species. J Biochem Biophys Methods. 2007;70(3):481–6. 10.1016/j.jbbm.2006.11.008 . 17196660

[25] Schwartz MW, Woods SC, Porte D Jr., Seeley RJ, Baskin DG. Central nervous system control of food intake. Nature. 2000;404(6778):661–71. Epub 2000/04/15. 10.1038/35007534 . 10766253

[26] Liao GY, An JJ, Gharami K, Waterhouse EG, Vanevski F, Jones KR, et al Dendritically targeted Bdnf mRNA is essential for energy balance and response to leptin. Nat Med. 2012;18(4):564–71. Epub 2012/03/20. 10.1038/nm.2687 22426422 PMC3327556

[27] Lebrun B, Bariohay B, Moyse E, Jean A. Brain-derived neurotrophic factor (BDNF) and food intake regulation: a minireview. Auton Neurosci. 2006;126–127:30–8. Epub 2006/04/25. 10.1016/j.autneu.2006.02.027 . 16632412

[28] Cone RD. Studies on the physiological functions of the melanocortin system. Endocrine reviews. 2006;27(7):736–49. 10.1210/er.2006-0034 . 17077189

[29] Combs TP, Berg AH, Obici S, Scherer PE, Rossetti L. Endogenous glucose production is inhibited by the adipose-derived protein Acrp30. The Journal of clinical investigation. 2001;108(12):1875–81. 10.1172/JCI14120 11748271 PMC209474

[30] Emanuelli B, Vienberg SG, Smyth G, Cheng C, Stanford KI, Arumugam M, et al Interplay between FGF21 and insulin action in the liver regulates metabolism. The Journal of clinical investigation. 2014;124(2):515–27. 10.1172/JCI67353 24401271 PMC3904602

[31] Xu J, Lloyd DJ, Hale C, Stanislaus S, Chen M, Sivits G, et al Fibroblast growth factor 21 reverses hepatic steatosis, increases energy expenditure, and improves insulin sensitivity in diet-induced obese mice. Diabetes. 2009;58(1):250–9. 10.2337/db08-0392 18840786 PMC2606881

[32] Camporez JP, Jornayvaz FR, Petersen MC, Pesta D, Guigni BA, Serr J, et al Cellular mechanisms by which FGF21 improves insulin sensitivity in male mice. Endocrinology. 2013;154(9):3099–109. 10.1210/en.2013-1191 23766126 PMC3749479

[33] Bjorbak C, Lavery HJ, Bates SH, Olson RK, Davis SM, Flier JS, et al SOCS3 mediates feedback inhibition of the leptin receptor via Tyr985. The Journal of biological chemistry. 2000;275(51):40649–57. 10.1074/jbc.M007577200 . 11018044

[34] D'Asti E, Long H, Tremblay-Mercier J, Grajzer M, Cunnane SC, Di Marzo V, et al Maternal dietary fat determines metabolic profile and the magnitude of endocannabinoid inhibition of the stress response in neonatal rat offspring. Endocrinology. 2010;151(4):1685–94. 10.1210/en.2009-1092 . 20160134

[35] Parente LB, Aguila MB, Mandarim-de-Lacerda CA. Deleterious effects of high-fat diet on perinatal and postweaning periods in adult rat offspring. Clinical nutrition. 2008;27(4):623–34. 10.1016/j.clnu.2008.05.005 . 18614261

[36] Ahima RS, Prabakaran D, Flier JS. Postnatal leptin surge and regulation of circadian rhythm of leptin by feeding. Implications for energy homeostasis and neuroendocrine function. The Journal of clinical investigation. 1998;101(5):1020–7. 10.1172/JCI1176 9486972 PMC508653

[37] Delahaye F, Breton C, Risold PY, Enache M, Dutriez-Casteloot I, Laborie C, et al Maternal perinatal undernutrition drastically reduces postnatal leptin surge and affects the development of arcuate nucleus proopiomelanocortin neurons in neonatal male rat pups. Endocrinology. 2008;149(2):470–5. 10.1210/en.2007-1263 . 18006626

[38] Bouret SG, Draper SJ, Simerly RB. Formation of projection pathways from the arcuate nucleus of the hypothalamus to hypothalamic regions implicated in the neural control of feeding behavior in mice. The Journal of neuroscience: the official journal of the Society for Neuroscience. 2004;24(11):2797–805. 10.1523/JNEUROSCI.5369-03.2004 . 15028773 PMC6729527

[39] Coupe B, Bouret SG. Development of the hypothalamic melanocortin system. Front Endocrinol (Lausanne). 2013;4:38 10.3389/fendo.2013.00038 23543895 PMC3608914

[40] Baquero AF, de Solis AJ, Lindsley SR, Kirigiti MA, Smith MS, Cowley MA, et al Developmental switch of leptin signaling in arcuate nucleus neurons. The Journal of neuroscience: the official journal of the Society for Neuroscience. 2014;34(30):9982–94. 10.1523/JNEUROSCI.0933-14.2014 25057200 PMC4107412

[41] Attig L, Solomon G, Ferezou J, Abdennebi-Najar L, Taouis M, Gertler A, et al Early postnatal leptin blockage leads to a long-term leptin resistance and susceptibility to diet-induced obesity in rats. Int J Obes (Lond). 2008;32(7):1153–60. 10.1038/ijo.2008.39 . 18379577

[42] Benoit C, Ould-Hamouda H, Crepin D, Gertler A, Amar L, Taouis M. Early leptin blockade predisposes fat-fed rats to overweight and modifies hypothalamic microRNAs. The Journal of endocrinology. 2013;218(1):35–47. 10.1530/JOE-12-0561 . 23576026

[43] Aalinkeel R, Srinivasan M, Song F, Patel MS. Programming into adulthood of islet adaptations induced by early nutritional intervention in the rat. American journal of physiology Endocrinology and metabolism. 2001;281(3):E640–8. . 11500321 10.1152/ajpendo.2001.281.3.E640

[44] Marcelino H, Veyrat-Durebex C, Summermatter S, Sarafian D, Miles-Chan J, Arsenijevic D, et al A role for adipose tissue de novo lipogenesis in glucose homeostasis during catch-up growth: a Randle cycle favoring fat storage. Diabetes. 2013;62(2):362–72. 10.2337/db12-0255 22961086 PMC3554390

[45] Plagemann A, Harder T, Rake A, Voits M, Fink H, Rohde W, et al Perinatal elevation of hypothalamic insulin, acquired malformation of hypothalamic galaninergic neurons, and syndrome x-like alterations in adulthood of neonatally overfed rats. Brain Res. 1999;836(1–2):146–55. . 10415413 10.1016/s0006-8993(99)01662-5

[46] Pedrazzi P, Cattaneo L, Valeriani L, Boschi S, Cocchi D, Zoli M. Hypothalamic neuropeptide Y and galanin in overweight rats fed a cafeteria diet. Peptides. 1998;19(1):157–65. . 9437748 10.1016/s0196-9781(97)00258-1

[47] Plaisance EP, Henagan TM, Echlin H, Boudreau A, Hill KL, Lenard NR, et al Role of beta-adrenergic receptors in the hyperphagic and hypermetabolic responses to dietary methionine restriction. Am J Physiol Regul Integr Comp Physiol. 2010;299(3):R740–50. 10.1152/ajpregu.00838.2009 20554934 PMC2944424

[48] Ueno N, Oh-ishi S, Segawa M, Nishida M, Fukuwatari Y, Kizaki T, et al Effect of age on brown adipose tissue activity in the obese (ob/ob) mouse. Mech Ageing Dev. 1998;100(1):67–76. . 9509396 10.1016/s0047-6374(97)00123-1

[49] Haynes WG, Morgan DA, Walsh SA, Mark AL, Sivitz WI. Receptor-mediated regional sympathetic nerve activation by leptin. The Journal of clinical investigation. 1997;100(2):270–8. 10.1172/JCI119532 9218503 PMC508189

[50] Enriori PJ, Sinnayah P, Simonds SE, Garcia Rudaz C, Cowley MA. Leptin action in the dorsomedial hypothalamus increases sympathetic tone to brown adipose tissue in spite of systemic leptin resistance. The Journal of neuroscience: the official journal of the Society for Neuroscience. 2011;31(34):12189–97. 10.1523/JNEUROSCI.2336-11.2011 21865462 PMC3758545

[51] Commins SP, Watson PM, Levin N, Beiler RJ, Gettys TW. Central leptin regulates the UCP1 and ob genes in brown and white adipose tissue via different beta-adrenoceptor subtypes. The Journal of biological chemistry. 2000;275(42):33059–67. 10.1074/jbc.M006328200 . 10938091

[52] Poher AL, Veyrat-Durebex C, Altirriba J, Montet X, D JC, Caillon A, et al Ectopic UCP1 overexpression in white adipose tissue improves insulin sensitivity in Lou/C rats, a model of obesity resistance. Diabetes. 2015 10.2337/db15-0210 . 26224884

